# Synergistic Associations of PNPLA3 I148M Variant, Alcohol Intake, and Obesity With Risk of Cirrhosis, Hepatocellular Carcinoma, and Mortality

**DOI:** 10.1001/jamanetworkopen.2022.34221

**Published:** 2022-10-03

**Authors:** Hyun-seok Kim, Xiangjun Xiao, Jinyoung Byun, Goo Jun, Stacia M. DeSantis, Han Chen, Aaron P. Thrift, Hashem B. El-Serag, Fasiha Kanwal, Christopher I. Amos

**Affiliations:** 1 Division of Gastroenterology and Hepatology, Beth Israel Deaconess Medical Center, Harvard Medical School, Boston, Massachusetts; 2 Section of Epidemiology and Population Sciences, Department of Medicine, Baylor College of Medicine, Houston, Texas; 3 Department of Epidemiology, Human Genetics & Environmental Sciences and Human Genetics Center, School of Public Health, The University of Texas Health Science Center at Houston, Houston; 4 Department of Biostatistics and Data Science, School of Public Health, The University of Texas Health Science Center at Houston, Houston; 5 Dan L. Duncan Comprehensive Cancer Center, Baylor College of Medicine, Houston, Texas; 6 Section of Gastroenterology and Hepatology, Baylor College of Medicine, Houston, Texas; 7 Clinical Epidemiology and Comparative Effectiveness Program, Section of Health Services Research, Michael E. DeBakey Veterans Affairs Medical Center, Houston, Texas; 8 Institute for Clinical and Translational Research, Baylor College of Medicine, Houston, Texas

## Abstract

**Importance:**

Alcohol drinking and obesity are associated with an increased risk of cirrhosis and hepatocellular carcinoma (HCC), but the risk is not uniform among people with these risk factors. Genetic variants, such as I148M in the patatin-like phospholipase domain-containing protein 3 (*PNPLA3*) gene, may play an important role in modulating cirrhosis and HCC risk.

**Objective:**

To investigate the joint associations of the *PNPLA3* I148M variant, alcohol intake, and obesity with the risk of cirrhosis, HCC, and liver disease–related mortality.

**Design, Setting, and Participants:**

This prospective cohort study analyzed 414 209 participants enrolled in the UK Biobank study from March 2006 to December 2010. Participants had no previous diagnosis of cirrhosis and HCC and were followed up through March 2021.

**Exposures:**

Self-reported alcohol intake (nonexcessive vs excessive), obesity (body mass index ≥30 [calculated as weight in kilograms divided by height in meters squared]), and *PNPLA3* I148M variant status (noncarrier, heterozygous carrier, or homozygous carrier) from initial assessment.

**Main Outcomes and Measures:**

The primary outcomes were incident cirrhosis and HCC cases and liver disease–related death ascertained from inpatient hospitalization records and death registry. The risks were calculated by Cox proportional hazards regression models.

**Results:**

A total of 414 209 participants (mean [SD] age, 56.3 [8.09] years; 218 567 women [52.8%]; 389 452 White race and ethnicity [94.0%]) were included. Of these participants, 2398 participants (0.6%) developed cirrhosis (5.07 [95% CI, 4.87-5.28] cases per 100 person-years), 323 (0.1%) developed HCC (0.68 [95% CI, 0.61-0.76] cases per 100 person-years), and 878 (0.2%) died from a liver disease–related cause (1.76 [95% CI, 1.64-1.88] cases per 100 person-years) during a median follow-up of 10.9 years. Synergistic interactions between the *PNPLA3* I148M variant, obesity, and alcohol intake were associated with the risk of cirrhosis, HCC, and liver disease–related mortality. The risk of cirrhosis increased supramultiplicatively (adjusted hazard ratio [aHR], 17.52; 95% CI, 12.84-23.90) in individuals with obesity, with excessive drinking, and who were homozygous carriers compared with those with no obesity, with nonexcessive drinking, and who were noncarriers. Supramultiplicative associations between the 3 factors and risks of HCC were found in individuals with 3 risk factors (aHR, 30.13; 95% CI, 16.51-54.98) and liver disease–related mortality (aHR, 21.82; 95% CI, 13.78-34.56). The *PNPLA3* I148M variant status significantly differentiated the risk of cirrhosis, HCC, and liver disease–related mortality in persons with excessive drinking and obesity.

**Conclusions and Relevance:**

This study found synergistic associations of the *PNPLA3* I148M variant, excessive alcohol intake, and obesity with increased risk of cirrhosis, HCC, and liver disease–related death in the general population. The *PNPLA3* I148M variant status may help refine the risk stratification for liver disease in persons with excessive drinking and obesity who may need early preventive measures.

## Key Points

**Questions:**

Can genotypic information about the patatin-like phospholipase domain-containing protein 3 (*PNPLA3*) I148M variant, a major genetic variant in liver diseases, play a role in stratifying the risk of individuals with alcohol consumption and obesity?

**Findings:**

In this cohort study of 414 209 participants in the UK Biobank study, the *PNPLA3* I148M variant had synergistic associations with the risk of cirrhosis, hepatocellular carcinoma, and liver disease–related mortality along with excessive alcohol intake and obesity.

**Meaning:**

Findings of this study suggest that genotyping for the *PNPLA3* I148M variant may be useful in refining the risk stratification of persons with obesity and excessive drinking who are at risk for advanced liver disease progression and may be candidates for early preventive measures.

## Teaser

This cohort study assesses the association of behavioral risk factors and genetic variants with risk of liver disease–related morbidity and mortality among middle-aged individuals.

## Introduction

Cirrhosis predisposes individuals to a range of complications, including ascites, hepatic encephalopathy, variceal bleeding, and hepatocellular carcinoma (HCC). Hepatocellular carcinoma is the seventh most frequently occurring cancer and the second most common cause of cancer mortality in the world.^1^ Appropriate screening and risk stratification for cirrhosis and HCC are important steps toward reducing liver disease–related morbidity and mortality. However, oversurveillance of individuals with low risk of morbidity and mortality and missed opportunities in individuals with high risk present substantial clinical challenges,^2^ highlighting a need for accurate methods of risk stratification for cirrhosis and HCC.

Alcohol drinking and obesity are important behavioral risk factors for cirrhosis and HCC.^3^ Consumption of more than 14 alcoholic drinks per week (40 g of alcohol per day) in men and more than 7 alcoholic drinks per week (20 g of alcohol per day) in women over a period of 10 years has been associated with a substantially increased risk of cirrhosis.^4,5^ Obesity can play a role in nonalcoholic steatohepatitis, leading to liver decompensation, HCC, and death.^6,7^ Yet, both alcohol drinking and obesity are common. The risk of cirrhosis and HCC in people with heavy alcohol intake and obesity also varies considerably, contributing to the difficulty of developing a clinically useful risk stratification strategy.

Genetic factors may play important roles in the complex pathogenesis of liver diseases and hence explain some of the variations in the risk of cirrhosis and HCC among people with heavy alcohol intake or obesity. Genetic association with cirrhosis and HCC among people without viral hepatitis has been reported for the rs738409 C>G single-nucleotide variant (I148M) in the patatin-like phospholipase domain-containing protein 3 (*PNPLA3*) gene (OMIM 609567) in chromosome 22.^8^ The *PNPLA3* I148M variant could modulate the outcomes of alcohol intake and obesity, but its interplay with these 2 risk factors in advanced liver disease remains unclear because of the lack of well-powered longitudinal studies.^9^

In this study, we aimed to investigate the joint associations of the *PNPLA3* I148M variant, alcohol intake, and obesity with the risk of cirrhosis, HCC, and liver disease–related mortality. We assessed participants in the UK Biobank on whether *PNPLA3* I148M variant status could help stratify the risk of heavy alcohol drinking and obesity.

## Methods

This cohort study was approved by the Baylor College of Medicine Institutional Review Board, which waived the informed consent requirement because of the use of deidentified data. We followed the Strengthening the Reporting of Observational Studies in Epidemiology (STROBE) reporting guideline.^10^

### Data Source and Study Population

UK Biobank is a large, ongoing prospective cohort study that recruited participants aged 40 to 69 years from 22 assessment centers throughout the UK from March 2006 to December 2010. Extensive health-related records were collected from these participants through questionnaires, electronic health records, and biological samples.^11,12^ We downloaded the UK Biobank data in October 2021.

**Figure 1.**
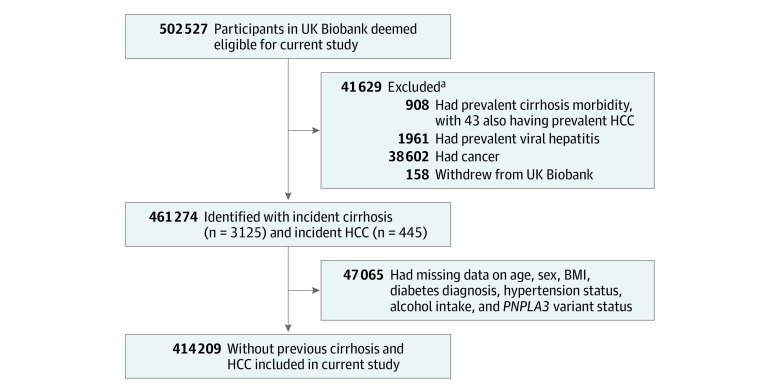
Participant Flowchart BMI indicates body mass index; HCC, hepatocellular carcinoma; and *PNPLA3*, patatin-like phospholipase domain-containing protein 3 gene. ^a^Some participants met multiple exclusion criteria.

Participants in the UK Biobank were eligible for inclusion in the present analysis. We excluded participants with prevalent cirrhosis and HCC that were recorded before or within 1 year of enrollment in the UK Biobank (Figure 1) to minimize misclassification incident or prevalent bias. In addition, we excluded participants with viral hepatitis to focus on those at risk for cirrhosis owing to the more common alcoholic or nonalcoholic fatty liver disease. Participants with nonliver cancers according to self-reported diagnosis and records from inpatient hospitalization were also excluded because they may have a high risk of mortality in the short term. People who withdrew from the UK Biobank and those with missing data at baseline for 1 or more key covariates were excluded.

### Primary and Secondary Outcomes

The primary outcomes were incident cases of cirrhosis and HCC and liver disease–related mortality recorded after 1 year of the UK Biobank enrollment through the end of the follow-up period. Hospitalizations for cirrhosis and HCC were identified by either primary or secondary diagnosis with specific *International Statistical Classification of Diseases and Related Health Problems, Tenth Revision* and Operations/Procedure Codes, Version 4 codes during any hospitalization, as previously described^13^ (eTable 1 in the Supplement). Secondary outcomes were overall and cardiovascular disease–related deaths.^13^ Liver disease–related and cardiovascular disease–related deaths were identified using *International Statistical Classification of Diseases and Related Health Problems, Tenth Revision* codes (eTable 2 in the Supplement).

Follow-up time for each outcome was calculated from 1 year after the date of UK Biobank enrollment until the date of cirrhosis or HCC diagnosis, death, or censoring (end of observation period or loss to follow-up), whichever occurred first. We censored follow-up at the date of hospital registry completion: March 31, 2021, for participants in England and Scotland, and February 28, 2018, for participants in Wales.

### Definition of Obesity and Alcohol Intake

Obesity was defined as body mass index (BMI) of 30 or higher, which was calculated as weight in kilograms divided by height in meters squared, at the baseline assessment visit. Alcohol intake was identified from a self-completed alcohol use questionnaire.

Participants were asked to report their mean alcohol intake per week or per month in terms of the number of glasses of red wine, champagne, or white wine; pints of beer or cider; measures of spirits; and glasses of fortified wine and other types of alcoholic drinks. Participants with nonweekly and occasional alcohol intake were asked to report consumption in a regular month to generate more reliable estimates for infrequent drinking. For each participant, the mean number of alcohol units consumed per week was calculated, assuming 2 units of pure alcohol in a pint of beer or cider; 1.5 units in a glass (125 mL) of red wine, champagne, white wine, fortified wine, and other alcoholic drink; and 1 unit in a measure (25 mL) of spirits.^14,15^

We categorized alcohol intake as excessive if the weekly intake reported was at least 36 units for men and at least 25 units for women. These thresholds represent the midpoint between hazardous and harmful drinking in the UK government guidelines.^16^

### Genotyping of the *PNPLA3* I148M Variant

The *PNPLA3* I148M variant was genotyped using either the UK BiLEVE Axiom Array or the UK Biobank Axiom Array, as previously reported.^12^ This information was coded as 0 for noncarriers (CC genotype), 1 for heterozygous carriers (GC genotype), and 2 for homozygous carriers (GG genotype) of the minor allele (G allele).

### Covariables

The covariables were age, sex, race and ethnicity, hypertension, type 2 diabetes, dyslipidemia, smoking status, and socioeconomic status. Age, sex, and race and ethnicity were self-reported. Race and ethnicity were categorized as a binary variable (White or others [Asian, Black, and mixed]) because 94% of participants in the UK Biobank were White individuals. 

Hypertension was identified according to self-reported history of hypertension, use of antihypertensive drugs, or systolic blood pressure higher than 140 mm Hg or diastolic blood pressure higher than 90 mm Hg, which was measured at the initial assessment. Dyslipidemia was identified according to self-reported history of dyslipidemia, use of cholesterol-lowering drugs, or total cholesterol level greater than 240 mg/dL (to convert to millimoles per liter, multiply by 0.0259), which was measured at the initial assessment. Type 2 diabetes was defined as self-reported history of type 2 or unspecified diabetes, current insulin treatment and/or use of oral hypoglycemic drugs, or fasting serum glucose level of 200 mg/dL (to convert to millimoles per liter, multiply by 0.0555) or hemoglobin A_1c_ level of at least 48 mmol/mol (6.5%), which was measured at the initial assessment. Details on how the UK Biobank collected and handled blood samples are described elsewhere.^17^ Briefly, fasting blood samples were collected at each local assessment center and shipped to the UK Biobank central laboratory for biomarker assays.

Smoking status was categorized as never, former, and current smoker according to a self-reported questionnaire. Socioeconomic status was derived from the Townsend index, a Census-based score of material deprivation based on the proportion of households without a car, overcrowded households, households that are not owner occupied, and unemployment in a given area.^18^ The Townsend index ranges from –6.26 to 11.0, with higher scores indicating a greater degree of deprivation.

### Statistical Analysis

The general characteristics of the study population were presented as descriptive statistics. We performed Cox proportional hazards regression models to evaluate the independent and joint associations of the *PNPLA3* I148M variant, obesity, and alcohol intake with time to cirrhosis, HCC, and liver disease–related death. Loss to follow-up, no event occurrence by end of study, and other causes of death were considered to be censoring events. We also examined overall and cardiovascular disease–related deaths as secondary outcomes. The survival package in R, version 4.0.2 (R Foundation for Statistical Computing) was used for the Cox proportional hazards regression models.^19^

We first investigated whether the 3 main risk factors (*PNPLA3* I148M variant, obesity, and alcohol intake) were associated with each of the primary outcomes, adjusting for covariates. Next, we estimated the adjusted hazard of each outcome between individuals with different combinations of risk factors (eg, no obesity, nonexcessive drinking, and noncarrier of the *PNPLA3* I148M variant; no obesity, nonexcessive drinking, and homozygous carrier of the *PNPLA3* I148M variant; obesity, nonexcessive drinking, and noncarrier of the *PNPLA3* I148M variant; no obesity, excessive drinking, and noncarrier of the *PNPLA3* I148M variant; or obesity, excessive drinking, and homozygous carrier of the *PNPLA3* I148M variant). The results were expressed as adjusted hazard ratios (aHRs) and 95% CIs.

In addition, to help guide clinical decision-making and policy, we estimated the cumulative 10-year incidence rate of primary outcomes by each combination of risk factors. We used the cmprsk package in R, version 4.0.2 (R Foundation for Statistical Computing), which estimates cumulative incidence rates with proportional subdistribution hazard regression models.^20^

We ran sensitivity and subgroup analyses stratified by sex to ascertain whether sex was an important effect modifier. We restricted the subgroup analyses to UK Biobank participants at risk for hepatic steatosis, defined as a Fatty Liver Index of 60 or higher.^21^ We focused on this subgroup because fatty liver is the most common cause of chronic liver disease. We also reran the primary analyses using a different definition of BMI (<25 as healthy, 25-30 as overweight, and ≥30 as obese) and alcohol intake (safe and hazardous consumption vs harmful consumption according to the UK guidelines^16^). A 2-sided *P* < .05 was considered to be statistically significant.

## Results

A total of 414 209 UK Biobank participants without previous diagnosis of cirrhosis and HCC were included in the present cohort study (Figure 1). Participants had a mean (SD) age of 56.3 (8.09) years and included 195 642 men (47.2%) and 218 567 women (52.8%); 94.0% identified as being of White race and ethnicity (Table 1). The proportion of current smokers was 10.5%, and the mean Townsend index was –1.33 (95% CI, −7.34 to 4.69). The proportion of individuals with hypertension was 56.1%, dyslipidemia was 52.1%, and diabetes was 6.0%. In total, 10.8% of participants reported excessive alcohol use and 23.8% reported obesity. There were 4.8% homozygous carriers of the *PNPLA3* I148M variant, and 21.6% had a minor allele (G allele). In those with excessive drinking and obesity, the prevalence of homozygous carriers was 4.7%.

**Table 1.** Demographic and Clinical Factors of Participants

Characteristic	Participants, No. (%)
Total No.	414 209
Age, mean (SD), y	56.3 (8.09)
Sex	
Male	195 642 (47.2)
Female	218 567 (52.8)
Race and ethnicity^a^	
White	389 452 (94.0)
Others^b^	24 757(6.0)
Smoking status	
Never	225 673 (54.5)
Former	143 577 (34.7)
Current	43 505 (10.5)
Missing data	1454 (0.3)
Alcohol intake	
Nonexcessive	369 616 (89.2)
Excessive	44 593 (10.8)
Townsend index, mean (95% CI)^c^	−1.33 (−7.34 to 4.69)
Hypertension	232 267 (56.1)
Dyslipidemia	215 767 (52.1)
Diabetes	24 921 (6.0)
Obesity	98 504 (23.8)
Follow-up time, mean (95% CI), mo	132 (92.4 to 169.9)
*PNPLA3* I148M variant status	
Noncarrier	254 927 (61.5)
Heterozygous carrier	139 570 (33.7)
Homozygous carrier	19 712 (4.8)

Abbreviation: *PNPLA3*, patatin-like phospholipase domain-containing protein 3 gene.

^a^ Race and ethnicity were self-reported and categorized as a binary variable (White or others) because most participants in the UK Biobank were White individuals.

^b^ Others included Asian, Black, and mixed.

^c^ Townsend index ranges from –6.26 to 11.0, with higher scores indicating a greater degree of deprivation.

During a median follow-up of 10.9 years, 2398 participants (0.6%) developed cirrhosis (5.07 [95% CI, 4.87-5.28] cases per 100 person-years), 323 (0.1%) developed HCC (0.68 [95% CI, 0.61-0.76] cases per 100 person-years), and 878 (0.2%) died from a liver disease–related cause (1.76 [95% CI, 1.64-1.88] cases per 100 person-years). Among 323 incident HCC cases, 169 (52.3%) had a previous diagnosis of cirrhosis, and 699 liver disease–related deaths (79.6%) were preceded by cirrhosis outcome.

### Factors Associated With Risk of Cirrhosis, HCC, and Liver Disease–Related Death

Table 2 describes the results of Cox proportional hazards regression models for the 3 primary outcomes. Compared with individuals who did not develop cirrhosis, those who developed cirrhosis tended to be older, male, and White individuals with lower socioeconomic status. They were also likely to report current smoking status and excessive alcohol intake, with multiple metabolic traits, such as hypertension, dyslipidemia, type 2 diabetes, and obesity. Similar patterns were observed in participants with HCC and liver disease–related deaths. Compared with variant noncarriers, the heterozygous (aHR, 1.40; 95% CI, 1.28-1.53) and homozygous (aHR, 2.45; 95% CI, 2.12-2.83) variant carriers had higher risks of progression to cirrhosis. The risk of cirrhosis was higher in those who reported excessive drinking (aHR, 2.32; 95% CI, 2.10-2.55) than in those with nonexcessive drinking. Obesity was also associated with risk of progression to cirrhosis (aHR, 1.85; 95% CI, 1.70-2.02).

**Table 2.** Factors Associated With Primary Outcomes

Factor	aHR (95% CI)^a^		
	Incident cirrhosis	Incident HCC	Liver disease–related death
No. of cases	2398	323	878
Age, per year	1.04 (1.03-1.05)	1.11 (1.09-1.13)	1.04 (1.03-1.05)
Sex			
Female	1 [Reference]	1 [Reference]	1 [Reference]
Male	1.55 (1.41-1.69)	2.79 (2.13-3.67)	2.47 (2.09-2.91)
Race and ethnicity^b^			
White	1.38 (1.13-1.69)	2.20 (1.08-4.49)	1.66 (1.15-2.38)
Others^c^	1 [Reference]	1 [Reference]	1 [Reference]
Smoking status			
Never	1 [Reference]	1 [Reference]	1 [Reference]
Former	1.14 (1.04-1.25)	1.36 (1.05-1.75)	1.21 (1.02-1.42)
Current	2.05 (1.82-2.30)	1.80 (1.27-2.58)	2.65 (2.20-3.19)
Alcohol intake			
Nonexcessive	1 [Reference]	1 [Reference]	1 [Reference]
Excessive	2.32 (2.10-2.55)	2.07 (1.58-2.70)	3.11 (2.68-3.60)
Townsend index^d^	1.08 (1.07-1.09)	1.04 (1.01-1.08)	1.09 (1.07-1.12)
Hypertension	1.42 (1.28-1.57)	1.29 (0.97-1.72)	1.68 (1.41-2.00)
Dyslipidemia	1.08 (0.98-1.19)	0.82 (0.63-1.07)	0.87 (0.75-1.02)
Diabetes	2.89 (2.59-3.22)	4.83 (3.73-6.26)	2.90 (2.44-3.45)
Obesity	1.85 (1.70-2.02)	2.02 (1.59-2.57)	1.96 (1.69-2.27)
*PNPLA3* I148M variant status			
Noncarrier	1 [Reference]	1 [Reference]	1 [Reference]
Heterozygous carrier	1.40 (1.28-1.53)	1.47 (1.15-1.87)	1.43 (1.24-1.65)
Homozygous carrier	2.45 (2.12-2.83)	4.38 (3.18-6.04)	2.67 (2.12-3.36)

Abbreviations: aHR, adjusted hazard ratio; HCC, hepatocellular carcinoma; *PNPLA3*, patatin-like phospholipase domain-containing protein 3 gene.

^a^ Adjusted for age; sex; race and ethnicity; smoking status; alcohol intake; Townsend index; hypertension, dyslipidemia, diabetes, or obesity; and *PNPLA3* I148M variant.

^b^ Race and ethnicity were self-reported and categorized as a binary variable (White or others) because most participants in the UK Biobank were White individuals.

^c^ Others included Asian, Black, and mixed.

^d^ Townsend index ranges from –6.26 to 11.0, with higher scores indicating a greater degree of deprivation.

Similar associations were observed for HCC and liver disease–related death. Homozygous variant carriers had a higher risk of HCC (aHR, 4.38; 95% CI, 3.18-6.04) and liver disease–related death (aHR, 2.67; 95% CI, 2.12-3.36) than variant noncarriers. However, the *PNLPA3* I148M variant was not associated with overall or cardiovascular disease–related deaths (eTable 3 in the Supplement).

### Synergistic Association 

**Figure 2.**
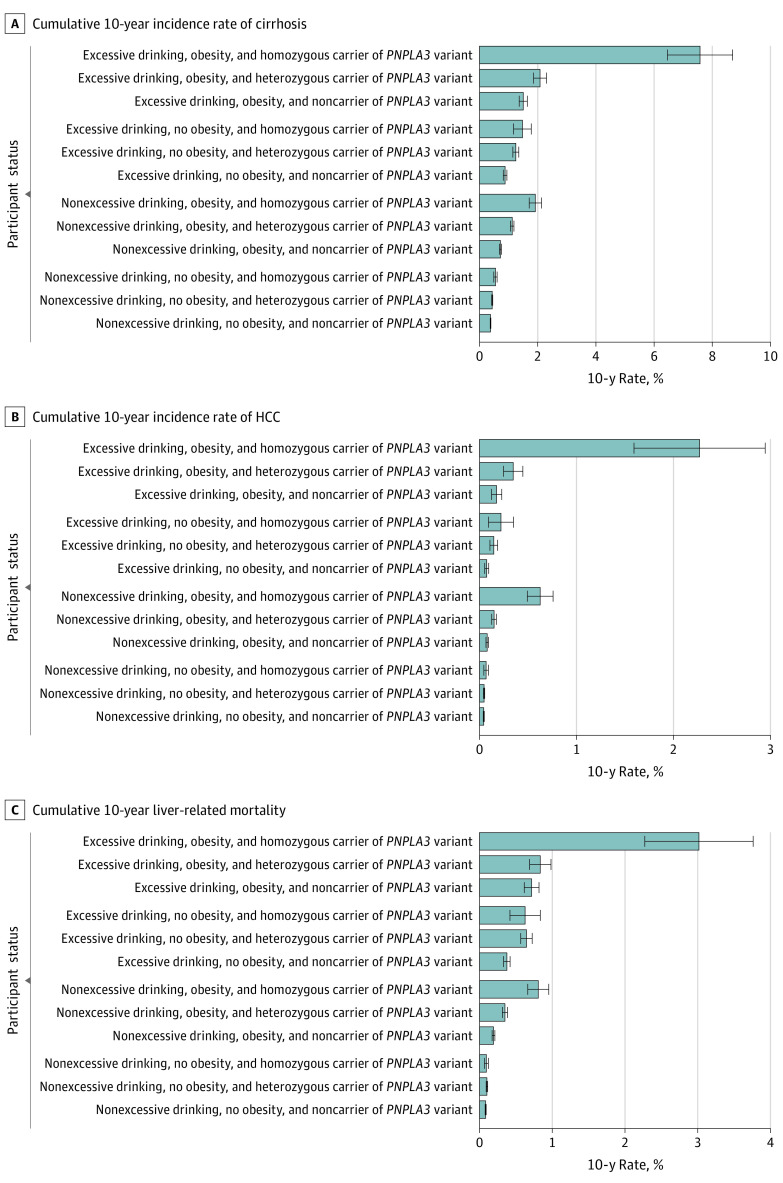
Cumulative 10-Year Incidence Rates by *PNPLA3* I148M Variant Status, Obesity, and Alcohol Intake Cumulative rate of each outcome was calculated according to the actual incidence of the outcome within each combination of risk factors, accounting for censoring. HCC indicates hepatocellular carcinoma; *PNPLA3*, patatin-like phospholipase domain-containing protein 3 gene.

In multivariable-adjusted Cox proportional hazards regression models that contained each possible combination of the 3 risk factors, we observed synergistic interactions between the *PNPLA3* I148M variant, obesity, and excessive alcohol intake that were associated with the risk of incident cirrhosis, HCC, or liver disease–related death (Table 3; Figure 2A). For example, among persons with no obesity and nonexcessive drinking, the risk of progression to cirrhosis was higher in homozygous variant carriers (aHR, 1.75; 95% CI, 1.36-2.24) than in noncarriers. Compared with individuals without any of the 3 risk factors, the risk of progression to cirrhosis was higher in those with only 1 risk factor (obesity: aHR, 1.76 [95% CI, 1.54-2.02]; excessive drinking: aHR, 2.35 [95% CI, 1.97-2.79]). The risk increased supramultiplicatively in individuals with obesity and excessive drinking who were homozygous variant carriers (aHR, 17.52; 95% CI, 12.84-23.90).

**Table 3.** Joint Association of *PNPLA3* I148M Variant, Alcohol Intake, and Obesity With Risk of Cirrhosis, HCC, and Liver Disease–Related Mortality

Risk factor	Total No.	Incident cirrhosis		Incident HCC		Liver disease–related death	
		No. of cases	aHR (95% CI)^**a**^	No. of cases	aHR (95% CI)^**a**^	No. of cases	aHR (95% CI)^**a**^
**Nonexcessive drinking**							
No obesity and *PNPLA3* I148M variant noncarrier	169 108	517	1 [Reference]	72	1 [Reference]	162	1 [Reference]
No obesity and *PNPLA3* I148M variant heterozygous carrier	93 587	349	1.23 (1.07-1.41)	45	1.12 (0.77-1.63)	104	1.17 (0.91-1.50)
No obesity and *PNPLA3* I148M variant homozygous carrier	13 309	72	1.75 (1.36-2.24)	9	1.57 (0.78-3.14)	16	1.26 (0.76-2.11)
**Obesity**							
Plus *PNPLA3* I148M variant noncarrier	54 051	382	1.76 (1.54-2.02)	46	1.40 (0.96-2.06)	125	1.73 (1.36-2.20)
Plus *PNPLA3* I148M variant heterozygous carrier	28 850	324	2.80 (2.43-3.23)	43	2.43 (1.65-3.59)	109	2.84 (2.21-3.65)
Plus *PNPLA3* I148M variant homozygous carrier	3990	78	4.81 (3.77-6.12)	25	9.63 (6.01-15.4)	37	6.85 (4.75-9.87)
**Excessive drinking**							
No obesity and *PNPLA3* I148M variant noncarrier	20 097	179	2.35 (1.97-2.79)	14	1.32 (0.74-2.35)	83	2.93 (2.24-3.83)
No obesity and *PNPLA3* I148M variant heterozygous carrier	10 738	137	3.42 (2.83-4.14)	17	3.04 (1.79-5.19)	76	5.11 (3.87-6.74)
No obesity and *PNPLA3* I148M variant homozygous carrier	1481	27	4.80 (3.25-7.07)	4	5.06 (1.84-13.9)	14	6.67 (3.86-11.6)
**Obesity**							
Plus *PNPLA3* I148M variant noncarrier	7120	113	3.57 (2.90-4.40)	14	2.86 (1.60-5.11)	57	4.85 (3.56-6.60)
Plus *PNPLA3* I148M variant heterozygous carrier	3947	88	4.86 (3.86-6.11)	13	4.36 (2.39-7.94)	42	6.22 (4.40-8.79)
Plus *PNPLA3* I148M variant homozygous carrier	546	44	17.52 (12.84-23.90)	13	30.13 (16.51-54.98)	21	21.82 (13.78-34.56)

Abbreviations: aHR, adjusted hazard ratio; HCC, hepatocellular carcinoma; *PNPLA3*, patatin-like phospholipase domain-containing protein 3 gene.

^a^ Adjusted for age; sex; race and ethnicity; smoking status; Townsend index; and hypertension, diabetes, or dyslipidemia.

We found similar synergistic associations between the *PNPLA3* I148M variant, obesity, and alcohol intake in the risk of progression to HCC and liver disease–related mortality (Table 3; Figure 2B and C). Compared with individuals without any of the 3 risk factors, the risk of HCC was higher in individuals with all risk factors (aHR, 30.13; 95% CI, 16.51-54.98). This risk was greater than the combination of the risks in persons with no obesity and nonexcessive drinking who were homozygous variant carriers (aHR, 1.57; 95% CI, 0.78-3.14), persons with obesity and nonexcessive drinking who were variant noncarriers (aHR, 1.40; 95% CI, 0.96-2.06), and persons with no obesity and with excessive drinking who were variant noncarriers (aHR, 1.32; 95% CI, 0.74-2.35).

The risk of liver disease–related mortality was higher in individuals with obesity and with excessive drinking who were homozygous variant carriers (aHR, 21.82; 95% CI, 13.78-34.56). This risk was greater than the combination of the risks in persons with no obesity and nonexcessive drinking who were homozygous variant carriers (aHR, 1.26; 95% CI, 0.76-2.11), persons with obesity and nonexcessive drinking who were variant noncarriers (aHR, 1.73; 95% CI, 1.36-2.20), and persons with no obesity and with excessive drinking who were variant noncarriers (aHR, 2.93; 95% CI, 2.24-3.83).

Figure 2 shows the cumulative 10-year risk of cirrhosis, HCC, and liver disease–related death in individuals with different combinations of obesity, excessive alcohol intake, and genetic variant. The 10-year risk of progression to cirrhosis was 0.28% (95% CI, 0.25%-0.31%) in persons without any of the 3 risk factors. This risk increased to 7.90% (95% CI, 5.57%-10.2%) in persons with excessive drinking and obesity who were homozygous variant carriers (Figure 2A) and decreased in between these 2 extremes for other groups. We found similar patterns in the cumulative incidence of HCC and liver disease–related mortality (Figure 2B and C). The 10-year risk of progression to HCC was 2.16% (95% CI, 0.90%-3.42%), whereas the 10-year liver disease–related mortality was 2.93% (95% CI, 1.51%-4.35%) in individuals with 3 risk factors. We did not observe any supramultiplicative associations between the *PNPLA3* I148M variant, obesity, and alcohol intake in overall and cardiovascular disease–related deaths (eTable 4 in the Supplement). However, participants with all 3 risk factors had higher risks than those with fewer risk factors.

### Sensitivity Analyses

The supramultiplicative interactive associations between the *PNPLA3* I148M variant, obesity, and alcohol intake persisted across sensitivity analyses, which included stratification by sex (eTable 5 in the Supplement), were restricted to individuals at risk for steatosis (eTable 6 in the Supplement), and used a different criteria of alcohol intake (eTable 7 in the Supplement) and BMI (eTable 8 in the Supplement). Among people at risk for steatosis, the cumulative 10-year risks were 8.24% (95% CI, 5.76%-10.7%) for cirrhosis and 2.37% (95% CI, 0.98%-3.76%) for HCC.

## Discussion

The public health burden of cirrhosis and HCC is expected to increase given the greater prevalence of excessive drinking and metabolic-associated fatty liver disease.^22^ Although alcohol intake and obesity are major risk factors for cirrhosis and HCC, the magnitude of risk is not uniform among people with these risks, suggesting that additional factors, including genetic variants, play a role. Results of this study suggest that the *PNPLA3* I148M variant, obesity, and alcohol intake interact synergistically and are associated with increased risk for cirrhosis, HCC, and liver disease–related death. Individuals with all 3 risk factors had 17.5-fold to 30.1-fold higher risk of progression to liver disease complications. We did not observe the synergistic interplay in overall and cardiovascular disease–related mortality. These synergistic associations with risk for liver–related diseases remain when limiting outcomes to participants at risk for steatosis, now the most common cause of chronic liver diseases. The results suggest that screening for obesity, heavy alcohol drinking, and a single genetic variant could distinguish people at risk for progressive liver disease with nonviral etiologies from people at low risk, particularly in the context of population screening programs. In addition, these 3 factors may be important to examine in prevention trials targeting individuals at high risk for progression to decompensated cirrhosis or HCC.

These findings are not only consistent with results of previous studies but also extend them.^23,24,25^ Analyzing the Dallas Heart Study and the Dallas Biobank, the Copenhagen City Heart Study, and the Copenhagen General Population Study populations, Stender et al^23^ reported a synergistic interaction between BMI and nonalcoholic fatty liver disease–related single-nucleotide variants (*PNPLA3* I148M, *TM6SF2* E167K, and *GCKR* P446L) in affecting hepatic triglyceride content, liver enzymes, and cirrhosis. In their study, Stender et al^23^ found the number of cirrhosis cases to be small (384 cases) and did not account for follow-up time. Another study reported that increasing visceral fat content was a factor in augmented association of the *PNPLA*3 I148M variant with hepatic fat content in 1019 European American men and 1238 European American women in the Family Heart Study.^24^ In addition, Emdin et al^25^ found that a polygenic risk score, including 12 genetic variants, had a synergistic association with alcohol intake and obesity and was associated with an increased risk of cirrhosis cases in the Partners HealthCare Biobank. However, the study did not account for follow-up time to estimate the risk in a prospective manner and did not examine the risk of HCC or mortality.

### Strengths and Limitations

This study has some strengths. It provides a unique opportunity to prospectively and longitudinally investigate the roles of the *PNPLA3* I148M variant, obesity, and alcohol intake in the risk of cirrhosis, HCC, and liver disease–related mortality. The follow-up data for inpatient hospitalization of participants in the UK Biobank are updated periodically; therefore, the number of cirrhosis cases in the current analysis has more than doubled to approximately 4000 (3125 incident cases and 908 prevalent cases [Figure 1]) compared with previous studies of cirrhosis in this population.^26,27^ In addition, *PNPLA3* I148M variant testing is now available. Furthermore, the prevalence of homozygous variant carriers in this study was not small (4.8%), suggesting that the findings may have clinical implications.^28^

This study also has some limitations. It is possible that cirrhosis and HCC cases were not captured completely because we relied on *International Statistical Classification of Diseases and Related Health Problems, Tenth Revision* or Operations/Procedure Codes, Version 4 codes, resulting in underestimation of incidence rates. In addition, the UK Biobank data set did not include abdominal imaging or laboratory tests for viral hepatitis, autoimmune hepatitis, or hemochromatosis; thus, we could not identify the etiologies of cirrhosis and HCC. However, most cases of cirrhosis and HCC were likely to have alcoholic or nonalcoholic fatty liver disease given the low prevalence of other etiologies in the general population. We also performed subgroup analysis by restricting the population to those at risk for steatosis, and the results remained similar (eTable 6 in the Supplement). In addition, behavioral risk factors, such as obesity and alcohol intake, may change over time, altering the risk of progression to cirrhosis and HCC. Because the UK Biobank measured only baseline BMI and alcohol intake, we could not investigate the associations between temporal changes in these risk factors and study outcomes. Given that approximately 90% of the UK Biobank participants were of European descent, further validation studies are needed to scrutinize whether these synergistic interactions are observed in other populations with different genetic ancestries.

## Conclusions

The *PNPLA3* I148M variant showed synergistic interplay with heavy alcohol intake and obesity and was associated with increased risk of developing cirrhosis, HCC, and liver disease–related death in a middle-aged population. Therefore, *PNPLA3* I148M genotypic information may be informative to refine cirrhosis and HCC risk stratification in the growing populations with heavy alcohol intake and obesity. Additional studies are needed in populations with different genetic ancestry to validate the findings and to incorporate *PNPLA3* I148M variant information in surveillance and/or chemoprevention of liver diseases.

## Notes

Supplement. **eTable 1.** ICD 10 and OPCS 4 Codes to Define Cirrhosis and HCC **eTable 2.** ICD 10 of Liver-Related and Cardiovascular Deaths **eTable 3.** Factors Associated With Secondary Outcomes **eTable 4.** Overall and Cardiovascular Mortality by PNPLA3 Variant, Obesity and Alcohol Intake **eTable 5.** Joint Associations of PNPLA3 Variant, Alcohol Intake, and Obesity Stratified by Sex **eTable 6.** Joint Associations in People At Risk for Steatosis **eTable 7.** Joint Associations With a Different Definition of Alcohol Intake **eTable 8.** Joint Associations With a Different Classification of BMI
