# Supplementary material for: Synergistic Associations of PNPLA3 I148M Variant, Alcohol Intake, and Obesity With Risk of Cirrhosis, Hepatocellular Carcinoma, and Mortality
Source: JAMA Netw Open. 2022 Oct 3;5(10):e2234221. doi: 10.1001/jamanetworkopen.2022.34221 (PMC9530967; doi:10.1001/jamanetworkopen.2022.34221)
Supplement: Supplement. — eTable 1. ICD 10 and OPCS 4 Codes to Define Cirrhosis and HCC eTable 2. ICD 10 of Liver-Related and Cardiovascular Deaths eTable 3. Factors Associated With Secondary Outcomes eTable 4. Overall and Cardiovascular Mortality by PNPLA3 Variant, Obesity and Alcohol Intake eTable 5. Joint Associations of PNPLA3 Variant, Alcohol Intake, and Obesity Stratified by Sex eTable 6. Joint Associations in People At Risk for Steatosis eTable 7. Joint Associations With a Different Definition of Alcohol Intake eTable 8. Joint Associations With a Different Classification of BMI [file jamanetwopen-e2234221-s001.pdf]

Supplemental Online Content

Kim HS, Xiao X, Byun J, et al. Synergistic associations of PNPLA3 I148M variant, alcohol intake, and obesity with risk of cirrhosis, hepatocellular carcinoma, and mortality. *JAMA Netw Open*. 2022;5(10):e2234221. doi:10.1001/jamanetworkopen.2022.34221

eTable 1. ICD 10 and OPCS 4 Codes to Define Cirrhosis and HCC

eTable 2. ICD 10 of Liver-Related and Cardiovascular Deaths

eTable 3. Factors Associated With Secondary Outcomes

eTable 4. Overall and Cardiovascular Mortality by PNPLA3 Variant, Obesity and Alcohol Intake

eTable 5. Joint Associations of PNPLA3 Variant, Alcohol Intake, and Obesity Stratified by Sex

eTable 6. Joint Associations in People At Risk for Steatosis

eTable 7. Joint Associations With a Different Definition of Alcohol Intake

eTable 8. Joint Associations With a Different Classification of BMI

This supplemental material has been provided by the authors to give readers additional information about their work.

© 2022 Kim HS et al. *JAMA Network Open.*

eTable 1. ICD 10 and OPCS 4 Codes to Define Cirrhosis and HCC

Code type	Code	Description
ICD 10	C22.0	Hepatocellular carcinoma
	K70.2	Alcoholic fibrosis and sclerosis of liver
	K70.3	Alcoholic cirrhosis of liver
	K72.1	Chronic hepatic failure
	K74.0	Hepatic fibrosis
	K74.1	Hepatic sclerosis
	K74.6	Other and unspecified cirrhosis of liver
	K76.6	Portal hypertension
	K76.7	Hepatorenal syndrome
	I85.0	Oesophageal varices with bleeding
	I85.9	Oesophageal varices without bleeding
	I86.4	Gastric varices
	I98.2	Oesophageal varices in diseases classified elsewhere
	I98.3	Oesophageal varices with bleeding in diseases classified elsewhere
	R18.0*	Ascites
OPSC4	J06.1	Transjugular intrahepatic insertion of stent into portal vein
	J06.2	Transjugular intrahepatic insertion of stent graft into portal vein
	G10.4	Local ligation of varices of oesophagus
	G10.8	Other specified open operations on varices of oesophagus
	G10.9	Unspecified open operations on varices of oesophagus
	G14.4	Fibreoptic endoscopic injection sclerotherapy to varices of oesophagus
	G17.4	Endoscopic injection sclerotherapy to varices of oesophagus using rigid oesophagoscope
	G43.7	Fibreoptic endoscopic rubber band ligation of upper gastrointestinal tract varices
	T46.1*	Paracentesis abdominis for ascites
	T46.2*	Drainage of ascites

ICD-10 refers to International Classification of Disease version 10. OPCS4 refers to Operation/Procedure codes version 4. Those with R18.0 and T46.1 & T46.2 codes were considered to have cirrhosis if accompanied by at least one corroborating ICD code for chronic liver disease (K70-K77) to remove ascites from non-hepatic causes.

© 2022 Kim HS et al. *JAMA Network Open.*

eTable 2. ICD 10 of Liver-Related and Cardiovascular Deaths

Cause of death	ICD 10Code	Description
Liver-related		
	C22.0	Hepatocellular carcinoma
	K70.X	Alcoholic liver disease
	K71.X	Toxic liver disease
	K72.X	Hepatic failure, not elsewhere classified
	K73.X	Chronic hepatitis, not elsewhere classified
	K74.X	Fibrosis and cirrhosis of liver
	K75.X	Other inflammatory liver diseases
	K76.X	Other diseases of liver
	I85.0	Oesophageal varices with bleeding
	I85.9	Oesophageal varices without bleeding
	I86.4	Gastric varices
	I98.2	Oesophageal varices in diseases classified elsewhere
	I98.3	Oesophageal varices with bleeding in diseases classified elsewhere
Cardiovascular		
	I10-I15	Hypertensive diseases
	I20-I25	Ischemic heart diseases
	I26-I28	Pulmonary heart disease and diseases of pulmonary circulation
	I30-I52	Other forms of heart diseases
	I60-I69	Cerebrovascular diseases
	I70-I79	Diseases of arteries, arterioles and capillaries

© 2022 Kim HS et al. *JAMA Network Open.*

eTable 3. Factors Associated With Secondary Outcomes

	aHR for overall mortality [95% CI] N=25,698	P-value	aHR for cardiovascular mortality [95% CI] N=8,983	P-value
Age	1.11 [1.10-1.11]	<0.001	1.12 [1.12-1.12]	<0.001
Sex				
Female	REF	REF	REF	REF
Male	1.56 [1.51-1.60]	<0.001	2.11 [2.01-2.22]	<0.001
Race				
White	1.34 [1.26-1.43]	<0.001	1.06 [0.96-1.17]	0.23
Others	REF	REF	REF	REF
Smoking				
Never	REF	REF	REF	REF
Former	1.31 [1.27-1.35]	0.01	1.36 [1.29-1.43]	<0.001
Current	2.72 [2.62-2.82]	<0.001	2.89 [2.72-3.08]	<0.001
Alcohol intake				
Non-excessive	REF	REF	REF	REF
Excessive	1.15 [1.11-1.19]	<0.001	1.08 [1.01-1.15]	0.01
Townsend Index	1.06 [1.05-1.06]	<0.001	1.07 [1.07-1.08]	<0.001
Hypertension	1.18 [1.14-1.21]	<0.001	1.55 [1.47-1.64]	<0.001
Dyslipidemia	1.11 [1.08-1.14]	<0.001	1.39 [1.32-1.46]	<0.001
Diabetes	1.75 [1.68-1.81]	<0.001	2.30 [2.18-2.43]	<0.001
Obesity	1.18 [1.15-1.22]	<0.001	1.35 [1.29-1.42]	<0.001
PNPLA3 variant status				
Non-carrier (CC)	REF	REF	REF	REF
Heterozygous carrier (GC)	1.01 [0.98-1.04]	0.54	1.02 [0.97-1.06]	0.52
Homozygous carrier (GG)	1.05 [0.99-1.11]	0.12	1.07 [0.97-1.19]	0.16
Abbreviation: aHR- adjusted hazard ratio, 95% CI: 95% confidence interval				

© 2022 Kim HS et al. *JAMA Network Open.*

eTable 4. Overall and Cardiovascular Mortality by PNPLA3 Variant, Obesity and Alcohol Intake

	aHR for Overall mortality	aHR for Cardiovascular mortaliy
Normal drinkers, non-obese with PNPLA3 non carriers	REF	REF
Normal drinkers, non-obese with PNPLA3 variant heterozygous	1.00 [95% CI: 0.97-1.04]	1.03 [95% CI: 0.97-1.10]
Normal drinkers, non-obese with PNPLA3 variant **homozygous**	1.01 [95% CI: 0.93-1.09]	1.04 [95% CI: 0.90-1.19]
Normal drinkers, **obese** with PNPLA3 variant non-carriers	1.19 [95% CI: 1.14-1.24]	1.38 [95% CI: 1.30-1.47]
Normal drinkers, **obese** with PNPLA3 variant heterozygous	1.23 [95% CI: 1.17-1.29]	1.42 [95% CI: 1.31-1.52]
Normal drinkers, **obese** with PNPLA3 variant homozygous	1.30 [95% CI: 1.16-1.45]	1.41 [95% CI: 1.17-1.69]
**Excessive** drinkers, non-obese with PNPLA3 variant non-carriers	1.20 [95% CI: 1.14-1.27]	1.14 [95% CI: 1.03-1.25]
**Excessive** drinkers, non-obese with PNPLA3 variant heterozygous	1.18 [95% CI:1.09-1.27]	1.12 [95% CI: 0.98-1.28]
**Excessive** drinkers, non-obese with PNPLA3 variant homozygous	1.34 [95% CI: 1.12-1.61]	1.39 [95% CI: 1.03-1.89]
**Excessive** drinkers, **obese** with PNPLA3 variant non-carriers	1.26 [95% CI: 1.16-1.37]	1.42 [95% CI: 1.25-1.62]
**Excessive** drinkers, **obese** with PNPLA3 variant heterozygous	1.24 [95% CI: 1.12-1.39]	1.25 [95% CI: 1.05-1.49]
**Excessive** drinkers, **obese** with PNPLA3 variant **homozygous**	1.61 [95% CI: 1.26-2.06]	2.17 [95% CI: 1.54-3.06]
Abbreviation: aHR- adjusted hazard ratio, 95% CI: 95% confidence interval		

© 2022 Kim HS et al. *JAMA Network Open.*

Adjusted for age, sex, race, smoking, Townsend index, hypertension, diabetes, dyslipidemia

© 2022 Kim HS et al. *JAMA Network Open.*

eTable 5. Joint Associations of PNPLA3 Variant, Alcohol Intake, and Obesity Stratified by Sex

	**Male**	**Female**
Risk factors	aHR for incident cirrhosis [95% CI]	aHR for incident cirrhosis [95% CI]
Non-excessive drinkers, non-obese with PNPLA3 non-carriers	REF	REF
Non-excessive drinkers, non-obese with PNPLA3 variant heterozygous	1.26 [1.05-1.51]	1.19 [0.96-1.46]
Non-excessive drinkers, non-obese with PNPLA3 variant **homozygous**	1.90 [1.38-2.61]	1.54 [1.04-2.30]
Non-excessive drinkers, **obese** with PNPLA3 variant non-carriers	1.89 [1.59-2.27]	1.54 [1.24-1.91]
Non-excessive drinkers, **obese** with PNPLA3 variant heterozygous	2.93 [2.43-3.53]	2.56 [2.05-3.19]
Non-excessive drinkers, **obese** with PNPLA3 variant homozygous	4.98 [3.63-6.83]	4.44 [3.05-6.46]
**Excessive** drinkers, non-obese with PNPLA3 variant non-carriers	2.67 [2.18-3.27]	1.70 [1.19-2.44]
**Excessive** drinkers, non-obese with PNPLA3 variant heterozygous	3.65 [2.91-4.59]	3.07 [2.14-4.42]
**Excessive** drinkers, non-obese with PNPLA3 variant homozygous	5.64 [3.69-8.64]	2.79 [1.04-7.50]

© 2022 Kim HS et al. *JAMA Network Open.*

**Excessive** drinkers, **obese** with PNPLA3 variant non-carriers	3.93<br>[3.10-4.98]	2.95<br>[1.86-4.68]
**Excessive** drinkers, **obese** with PNPLA3 variant heterozygous	6.04<br>[4.71-7.75]	1.56<br>[0.69-3.52]
**Excessive** drinkers, **obese** with PNPLA3 variant **homozygous**	18.4<br>[13.0-26.1]	18.3<br>[9.04-37.2]

© 2022 Kim HS et al. *JAMA Network Open.*

eTable 6. Joint Associations in People At Risk for Steatosis

	aHR for cirrhosis	aHR for HCC	aHR for liver-related death
Non-excessive drinkers, non-obese with PNPLA3 non carriers	REF	REF	REF
Non-excessive drinkers, non-obese with PNPLA3 variant heterozygous	1.43 [95% CI: 1.16-1.77]	1.54 [95% CI: 0.89-2.66]	1.29 [95% CI: 0.91-1.83]
Non-excessive drinkers, non-obese with PNPLA3 variant **homozygous**	2.13 [95% CI: 1.47-3.10]	1.84 [95% CI: 0.64-5.24]	2.02 [95% CI: 1.10-3.71]
Non-excessive drinkers, **obese** with PNPLA3 variant non-carriers	1.16 [95% CI: 0.97-1.39]	1.17 [95% CI: 0.72-1.90]	1.05 [95% CI: 0.79-1.42]
Non-excessive drinkers, **obese** with PNPLA3 variant heterozygous	1.87 [95% CI: 1.55-2.25]	2.12 [95% CI: 1.31-3.44]	1.72 [95% CI: 1.27-2.33]
Non-excessive drinkers, **obese** with PNPLA3 variant homozygous	3.19 [95% CI: 2.42-4.20]	8.09 [95% CI: 4.63-14.1]	4.09 [95% CI: 2.72-6.15]
**Excessive** drinkers, non-obese with PNPLA3 variant non-carriers	2.54 [95% CI: 2.00-3.22]	1.51 [95% CI: 0.71-3.21]	3.03 [95% CI: 2.14-4.29]
**Excessive** drinkers, non-obese with PNPLA3 variant heterozygous	3.51 [95% CI: 2.70-4.56]	4.38 [95% CI: 2.30-8.35]	4.89 [95% CI: 3.39-7.04]
**Excessive** drinkers, non-obese with PNPLA3 variant homozygous	4.96 [95% CI: 3.02-8.16]	4.06 [95% CI: 0.97-17.1]	4.93 [95% CI: 2.27-10.7]
**Excessive** drinkers, **obese** with PNPLA3 variant non-carriers	2.44 [95% CI: 1.92-3.10]	2.09 [95% CI: 1.06-4.12]	2.86 [95% CI:2.00-4.11]

© 2022 Kim HS et al. *JAMA Network Open.*

**Excessive** drinkers, **obese** with PNPLA3 variant heterozygous	3.37 [95% CI: 2.60-4.37]	3.41 [95% CI: 1.72-6.73]	3.86 [95% CI: 2.61-5.69]
**Excessive** drinkers, **obese** with PNPLA3 variant **homozygous**	11.9 [95% CI: 8.50-16.7]	24.6 [95% CI: 12.7-47.8]	13.1 [95% CI:7.90-21.7]

© 2022 Kim HS et al. *JAMA Network Open.*

eTable 7. Joint Associations With a Different Definition of Alcohol Intake

	aHR for cirrhosis	aHR for HCC	aHR for liver-related death
**Safe** drinkers, non-obese with PNPLA3 non-homozygous carriers	REF	REF	REF
**Safe** drinkers, **obese** with PNPLA3 non-homozygous	1.98 [95% CI: 1.76-2.22]	1.58 [95% CI: 1.14-2.17]	2.25 [95% CI: 1.83-2.77]
**Safe** drinkers, non-obese with PNPLA3 variant **homozygous**	1.54 [95% CI: 1.16-2.03]	1.60 [95% CI: 0.78-3.28]	0.98 [95% CI: 0.52-1.85]
Safe drinkers, **obese** with PNPLA3 variant **homozygous**	4.90 [95% CI: 3.82-6.29]	8.60 [95% CI: 5.22-14.2]	7.55 [95% CI: 5.15-11.1]
**Hazardous** drinkers, non-obese with PNPLA3 non-homozygous carriers	1.18 [95% CI: 1.02-1.35]	0.88 [95% CI: 0.59-1.32]	1.54 [95% CI: 1.23-1.94]
**Hazardous** drinkers, **obese** with PNPLA3 non-homozygous	2.18 [95% CI: 1.85-2.56]	1.94 [95% CI: 1.27-2.97]	2.76 [95% CI: 2.12-3.60]
**Hazardous** drinkers, non-obese with PNPLA3 variant **homozygous**	1.66 [95% CI: 1.06-2.59]	1.13 [95% CI: 0.28-4.60]	2.10 [95% CI: 1.03-4.26]
**Hazardous** drinkers, **obese** with PNPLA3 variant **homozygous**	5.40 [95% CI: 3.62-8.07]	14.0 [95% CI: 7.45-26.4]	7.26 [95% CI: 3.94-13.4]
**Harmful** drinkers, non-obese with PNPLA3 non-homozygous carriers	3.93 [95% CI: 3.34-4.62]	2.64 [95% CI: 1.60-4.37]	5.84 [95% CI: 4.58-7.46]
**Harmful** drinkers, **obese** with PNPLA3 non-homozygous	5.09 [95% CI: 4.18-6.20]	4.15 [95% CI: 2.41-7.13]	7.11 [95% CI: 5.27-9.60]
**Harmful** drinkers, non-obese with PNPLA3 variant **homozygous**	9.40 [95% CI: 6.25-14.2]	8.10 [95% CI: 2.56-25.7]	13.0 [95% CI: 7.25-23.4]
**Harmful** drinkers, **obese** with PNPLA3 variant **homozygous**	22.2 [95% CI: 15.1-32.7]	32.9 [95% CI: 15.1-71.7]	34.5 [95% CI:20.3-58.6]

© 2022 Kim HS et al. *JAMA Network Open.*

eTable 8. Joint Associations With a Different Classification of BMI

	aHR for cirrhosis	aHR for HCC	aHR for liver-related death
Non-excessive drinkers, normal weight with PNPLA3 non-homozygous carriers	REF	REF	REF
Non-excessive drinkers, normal weight with PNPLA3 **homozygous carriers**	1.63 [95% CI: 1.08-2.45]	2.72 [95% CI: 1.07-6.97]	0.99 [95% CI: 0.40-2.44]
Non-excessive drinkers, **overweight** with PNPLA3 non-homozygous carriers	1.18 [95% CI: 1.02-1.36]	1.30 [95% CI: 0.86-1.95]	0.99 [95% CI: 0.77-1.28]
Non-excessive drinkers, **overweight** with PNPLA3 **homozygous carriers**	1.90 [95% CI: 1.39-2.60]	1.26 [95% CI: 0.44-3.55	1.28 [95% CI: 0.68-2.39]
Non-excessive drinkers, **obese** with PNPLA3 non-homozygous carriers	2.19 [95% CI: 1.89-2.53]	2.05 [95% CI: 1.34-3.11]	2.07 [95% CI: 1.61-2.67]
Non-excessive drinkers, **obese** weight with PNPLA3 **homozygous carriers**	4.96 [95% CI: 3.84-6.40]	11.1 [95% CI: 6.49-19.1]	6.59 [95% CI: 4.46-9.75]
**Excessive** drinkers, normal weight with PNPLA3 non-homozygous carriers	3.06 [95% CI: 2.45-3.81]	0.94 [95% CI: 0.33-2.66]	4.56 [95% CI: 3.32-6.26
**Excessive** drinkers, normal weight with PNPLA3 **homozygous carriers**	7.63 [95% CI: 4.46-13.1]	9.77 [95% CI: 2.34-40.8]	12.0 [95% CI: 6.05-23.9]
**Excessive** drinkers, **overweight** with PNPLA3 non-homozygous carriers	2.68 [95% CI: 2.23-3.22]	2.73 [95% CI: 1.63-4.59]	3.07 [95% CI: 2.29-4.13]

© 2022 Kim HS et al. *JAMA Network Open.*

**Excessive** drinkers, **overweight** with PNPLA3 **homozygous carriers**	3.58 [95% CI: 2.05-6.25]	4.13 [95% CI: 0.98-17.3]	3.42 [95% CI: 1.39-8.43]
**Excessive** drinkers, **obese** with PNPLA3 non-homozygous carriers	4.18 [95% CI: 3.46-5.04]	3.98 [95% CI: 2.34-6.76]	5.17 [95% CI: 3.85-6.95]
**Excessive** drinkers, **obese** weight with PNPLA3 **homozygous carriers**	18.1 [95% CI: 13.1-25.0]	35.0 [95% CI: 18.1-67.7]	20.6 [95% CI:12.7-33.4]

© 2022 Kim HS et al. *JAMA Network Open.*
